# Surgical Outcomes after Full Thickness Chest Wall Resection Followed by Immediate Reconstruction: A 7-Year Observational Study of 42 Cases

**DOI:** 10.1016/j.jpra.2024.04.006

**Published:** 2024-04-18

**Authors:** Cloë L. Sparreboom, M. Jenda Hop, Masood Mazaheri, Joost Rothbarth, Alexander P.W.M. Maat, Eveline M.L. Corten, Marc A.M. Mureau

**Affiliations:** a Department of Plastic and Reconstructive Surgery, Erasmus MC Cancer Institute, University Medical Center Rotterdam, Rotterdam, The Netherlands; b Department of Surgical Oncology and Gastrointestinal Surgery, Erasmus MC Cancer Institute, University Medical Center Rotterdam, Rotterdam, the Netherlands; c Department of Cardiothoracic Surgery, Erasmus MC, University Medical Center Rotterdam, Rotterdam, The Netherlands.

**Keywords:** Chest wall reconstruction, Chest wall tumors, Postoperative complications, Full thickness chest wall defect

## Abstract

**Introduction:**

Reconstruction of full thickness chest wall defects is challenging and is associated with a considerable risk of complications. Therefore, the aim of this study was to investigate the surgical outcomes and their associations with patient and treatment characteristics following full thickness chest wall reconstruction.

**Patients and methods:**

A retrospective observational study was performed by including patients who underwent reconstruction of full thickness chest wall defect at the Erasmus MC between January 2014 and December 2020. The type of reconstruction was categorized into skeletal and soft tissue reconstructions. For skeletal reconstruction, only non-rigid prosthetic materials were used. Patient and surgical characteristics were retrieved and analyzed for associations with postoperative complications.

**Results:**

Thirty-two women and 10 men with a mean age of 60 years were included. In 26 patients (61.9%), the reconstruction was performed using prosthetic material and a soft tissue flap, in nine cases (21.4%) only a soft tissue flap was used, and in seven other patients (16.7%) only the prosthetic material was used. Pedicled musculocutaneous latissimus dorsi flaps were used most often (n=17), followed by pectoralis major flaps (n=8) and free flaps (n=8). Twenty-two patients (52.4%) developed at least one postoperative complication. Wounds (21.4%) and pulmonary (19.0%) complications occurred most frequently. Five (11.9%) patients required reoperation. There were no associations between patient and treatment characteristics and the occurrence of major complications. There was no mortality.

**Conclusions:**

Reconstruction of full thickness chest wall defects using only non-rigid prosthetic material for skeletal reconstruction appears safe with an acceptable reoperation rate and low mortality, questioning the need for rigid fixation techniques.

## Introduction

Full thickness chest wall resection followed by immediate reconstruction is a challenging procedure with potentially high postoperative complication and mortality risk. The main goals of chest wall reconstruction are to maintain chest wall stability, preserve breathing and protect intrathoracic organs.^1^ The main indications for full thickness chest wall resections include metastatic or locally advanced breast cancer, radiation-induced malignancies and osteoradionecrosis. These oncologic chest wall resections often require extensive resection of skeletal structures along with the overlying soft tissues, including potential donor-site muscles for the reconstruction.^2^

Full thickness chest wall resections became established in the beginning of the 20^th^ century following the advancements in surgical treatment for tuberculosis.^3^, ^4^, ^5^ Since then, several techniques have been described for the reconstruction of chest wall defects. Historically, reconstruction was performed using fascia lata, rib or cutaneous grafts, but nowadays rigid and non-rigid prosthetic as well as biological materials are available.^6^ After restoring the structural stability of the bony thorax, soft tissue coverage can be achieved using predominantly pedicled musculocutaneous flaps such as the latissimus dorsi or pectoralis major flaps.^7^ In some cases, free flaps are used, if the pedicled flaps do not reach the defect or have failed previously.^8^

Despite advancements in surgical techniques and postoperative care, the complication rates after chest wall reconstruction still vary from 16% to as high as 62%,^1^^,^^6^^,^^9^, ^10^, ^11^, ^12^, ^13^, ^14^ with pulmonary complications being the most frequent.^6^ The other frequently encountered complication is compromised wound healing.^15^^,^^16^ However, the current knowledge on postoperative outcomes remains scarce. Likely because of the rarity of chest wall resections, and only a few studies exist with sample size larger than 100 patients.^1^^,^^10^^,^^12^, ^13^, ^14^^,^^16^ Most of these previous studies are based on heterogeneous patient populations, including partial and full thickness defects. Only one study from 1999 by Deschamps et al. included only full thickness defects.^12^ Furthermore, these previous studies reported general, mostly single-surgeon experience in chest wall resection without specifically investigating postoperative complications or associations between outcomes and patient and surgical characteristics.

Increasing our understanding of risk factors for postoperative complications by assessing the associations in a clear homogenous study population of only full thickness chest wall resections could result in better selection of surgical techniques and early complication detection and treatment, thereby improving postoperative outcomes.

Therefore, the aim of this study was to assess the surgical outcomes and investigate the potential associations between patient and treatment characteristics and postoperative complications following full thickness chest wall reconstruction.

## Materials and methods

### Study design

A retrospective observational study was performed according to the STROBE guidelines and included all consecutive patients with tumor or a history of tumor who underwent full thickness chest wall resection and immediate reconstruction at the Erasmus MC, University Medical Center Rotterdam between January 2014 and December 2020. A full thickness chest wall resection was defined as the resection of at least one rib or (part of) the sternum in combination with surgical removal of the overlying soft tissues. The decision on the type of reconstruction used was at the individual surgeon's discretion.

The present study was approved by the institutional review board of the Erasmus University Medical Center (MEC-2021-0331) and was conducted in accordance with the national legislation and declaration of Helsinki.

### Data collection

Electronic patient files were reviewed and data were collected on patient characteristics (sex, age, height, weight, American Society of Anesthesiologists (ASA) score, diabetes mellitus and smoking, neoadjuvant treatment and indication for surgery) and surgical characteristics (location, type of resection and reconstruction, operative time and defect surface area). The type of reconstruction was categorized into skeletal and soft tissue reconstructions. The Charlson Comorbidity Index was used to summarize each patient's comorbid burden.^17^

The primary outcome was the occurrence of any postoperative complication within 90 days after surgery. We defined a postoperative complication as any undesirable, unintended and direct result of an operation affecting the patient.^18^ Complications were grouped into wound, pulmonary, mesh-related, flap failure and non-surgical complications. Postoperative complications were grouped according to the Clavien–Dindo classification consisting of seven grades based on the severity and type of intervention needed.^19^ A Clavien–Dindo classification of ≥3 was considered a major complication. Secondary outcomes were length of hospital stay, readmissions, reoperations and 90-day mortality.

### Statistical analysis

Normality of continuous variables was tested using the Kolmogorov–Smirnov test. Continuous variables with a normal distribution are described as means with standard deviation (SD) and those without a normal distribution are presented as medians with interquartile ranges (IQRs). Dichotomous and categorical variables are expressed as numbers with percentages.

Student's *t*-tests and Mann–Whitney U tests were used to compare group means and medians between two groups, respectively. One way-ANOVA and Kruskall–Wallis *H* tests were used to compare group means and medians, respectively, between more than two groups. Fisher's exact and chi-square tests were used to analyze dichotomous or categorical variables. In addition, odds ratios were computed to analyze the strength of association between the variables. Two-sided *p* values less than 0.05 were considered statistically significant. All analyses were performed using IBM SPSS Statistics, version 25 (IBM, Armonk, NY).

## Results

Forty-two patients, 32 women and 10 men, with a mean age of 59.6 years were included (Table 1). The majority (71.4%) received neoadjuvant chemotherapy, radiotherapy or a combination of both (Table 2). Breast cancer was the most common indication for full thickness chest wall resection (50.0%). Seven patients in this subgroup had primary locally advanced cancer, and 14 had locally recurrent or metastatic cancer. Other indications were sarcoma, skin cancer, colorectal cancer, osteoradionecrosis or infection, intrathoracic tumors and paraganglioma.

**Table 1:** Characteristics of 42 patients with full thickness chest wall defects.

		Total (n = 42)	Missing*
Sex	Male	10 (23.8%)	
	Female	32 (76.2%)	
Age, mean (SD), years		59.6 (12.6)	
BMI, median (IQR), kg/m^2^		25.1 (22.8 – 28.3)	
ASA score	I	3 (7.1%)	1 (2.4%)
	II	30 (71.4%)	
	III	7 (16.7%)	
	IV	1 (2.4%)	
Diabetes mellitus		2 (4.8%)	
Current smoking		7 (16.7%)	4 (9.5%)
Neoadjuvant treatment	None	12 (28.6%)	
	Radiotherapy	7 (16.7%)	
	Chemotherapy	15 (35.7%)	
	Chemoradiotherapy	8 (19.0%)	
Charlson Comorbidity Index, median (IQR)		3 (2–5)	

SD, standard deviation; IQR, interquartile range; BMI, Body Mass Index; ASA, American Society of Anesthesiologists.

⁎ There were no statistically significant differences in sex, age, BMI, ASA, diabetes mellitus, neoadjuvant treatment and Charlson Comorbidity Index for patients with missing and without values in smoking.

**Table 2:** Indication for full thickness chest wall resection.

Indication	Type of tumor	Total (n = 42)
Breast cancer		21 (50.0%)
	*Primary (locally advanced)*	7
	*Recurrent/metastatic*	14
Sarcoma		8 (19.0%)
	*Primary*	7
	*Recurrent/metastatic*	1
Skin cancer		4 (9.5%)
	*Basal cell carcinoma*	3
	*Squamous cell carcinoma*	1
Colorectal cancer		2 (4.8%)
	*Primary (locally advanced)*	1
	*Recurrent/metastatic*	1
Others		7 (16.7%)
	*Osteoradionecrosis/infection*	4
	*Intrathoracic tumor*	2
	*Paraganglioma*	1

Most resections were located anterolaterally (78.6%; Table 3). At least one rib was resected in 35 patients (83.3%). The median (IQR) number of resected ribs was 2 (1-3). Reconstructions were categorized into skeletal and soft tissue reconstructions. In 33 patients (78.6%), skeletal reconstruction required the use of prosthetic materials. In 26 patients (61.9%), full thickness chest wall defect was reconstructed using prosthetic material and a soft tissue flap, in nine other patients (21.4%) with only a soft tissue flap and in seven cases (16.7%) with only prosthetic material. In the present study, only non-rigid skeletal reconstructions were performed using either a synthetic mesh (polypropylene, 90.9%; polytetrafluoroethylene, 3.0%) or a porcine, cross-linked acellular dermal matrix (6.1%). These acellular dermal matrices were predominantly used for larger, complex defects or after resection of ulcerating tumors because of better infection resistance. A pedicled musculocutaneous latissimus dorsi (LD) flap was most often used for soft tissue reconstruction. Microvascular free tissue transfer was required in eight only patients (19.0%).

**Table 3:** Chest wall resection and reconstruction characteristics.

		Total (n = 42)
**Resection**		
Location	Anterolateral	33 (78.6%)
	Anterior	5 (11.9%)
	Posterolateral	2 (4.8%)
	Thoracoabdominal	1 (2.4%)
	Forequarter amputation	1 (2.4%)
Skeletal resection	Rib(s) only	14 (33.3%)
	Rib(s) + partial sternum	14 (33.3%)
	Partial sternum	7 (16.7%)
	Rib(s) + lung	4 (9.5%)
	Rib(s) + clavicle	1 (2.4%)
	Rib(s) + partial sternum + clavicle	1 (2.4%)
	Rib(s) + partial sternum + lung	1 (2.4%)
**Reconstruction**		
Skeletal and soft tissue		26 (61.9%)
Soft tissue only		9 (21.4%)
Skeletal only		7 (16.7%)
		
Skeletal reconstruction	Mesh	33 (78.6%)
	No mesh	9 (21.4%)
Soft tissue reconstruction	Pedicled muscle flap	18 (42.9%)
	*LD*	17
	*Trapezius + Deltoid*	1
	Free flap	8 (19.0%)
	*LD*	2
	*VRAM*	2
	*TRAM*	1
	*TFL + VL*	1
	*DIEP*	1
	*ALT*	1
	Muscle advancement flap	8 (19.0%)
	*Unilateral PM*	6
	*Bilateral PM*	2
	Primary closure	7 (16.7%)
	Local transposition	1 (2.4%)

LD, latissimus dorsi; VRAM, vertical rectus abdominis muscle; TRAM, transverse rectus abdominis muscle; TFL + VL, tensor fasciae lata + vastus lateralis; DIEP, deep inferior epigastric perforator; ALT, anterolateral thigh; PM, pectoralis major.

**Figure 1:**
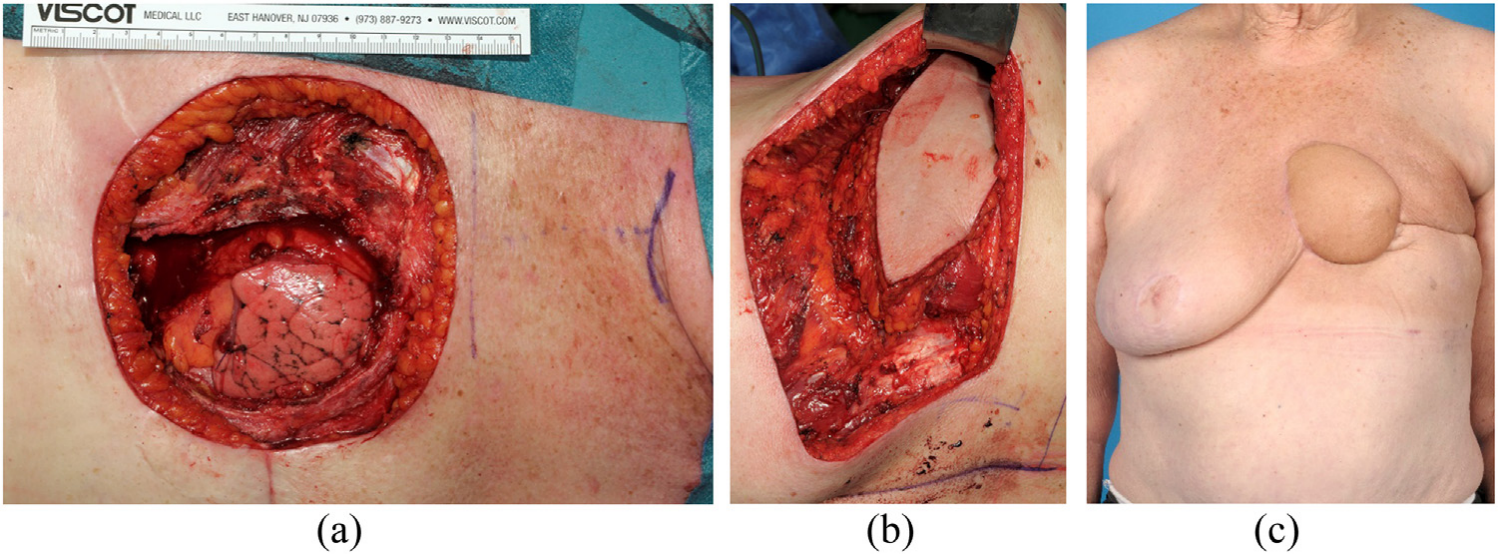
Intraoperative and postoperative photographs of a case with locally recurrent breast cancer which was treated by partial sternectomy and resection of the 3^rd^, 4^th^, and 5^th^ rib followed by reconstruction with a prolene mesh and a pedicled musculocutaneous latissimus dorsi flap. A. Intraoperative defect. B. Tranposition of the pedicled musculocutaneous latissimus dorsi flap. C. Postoperative.

**Figure 2:**
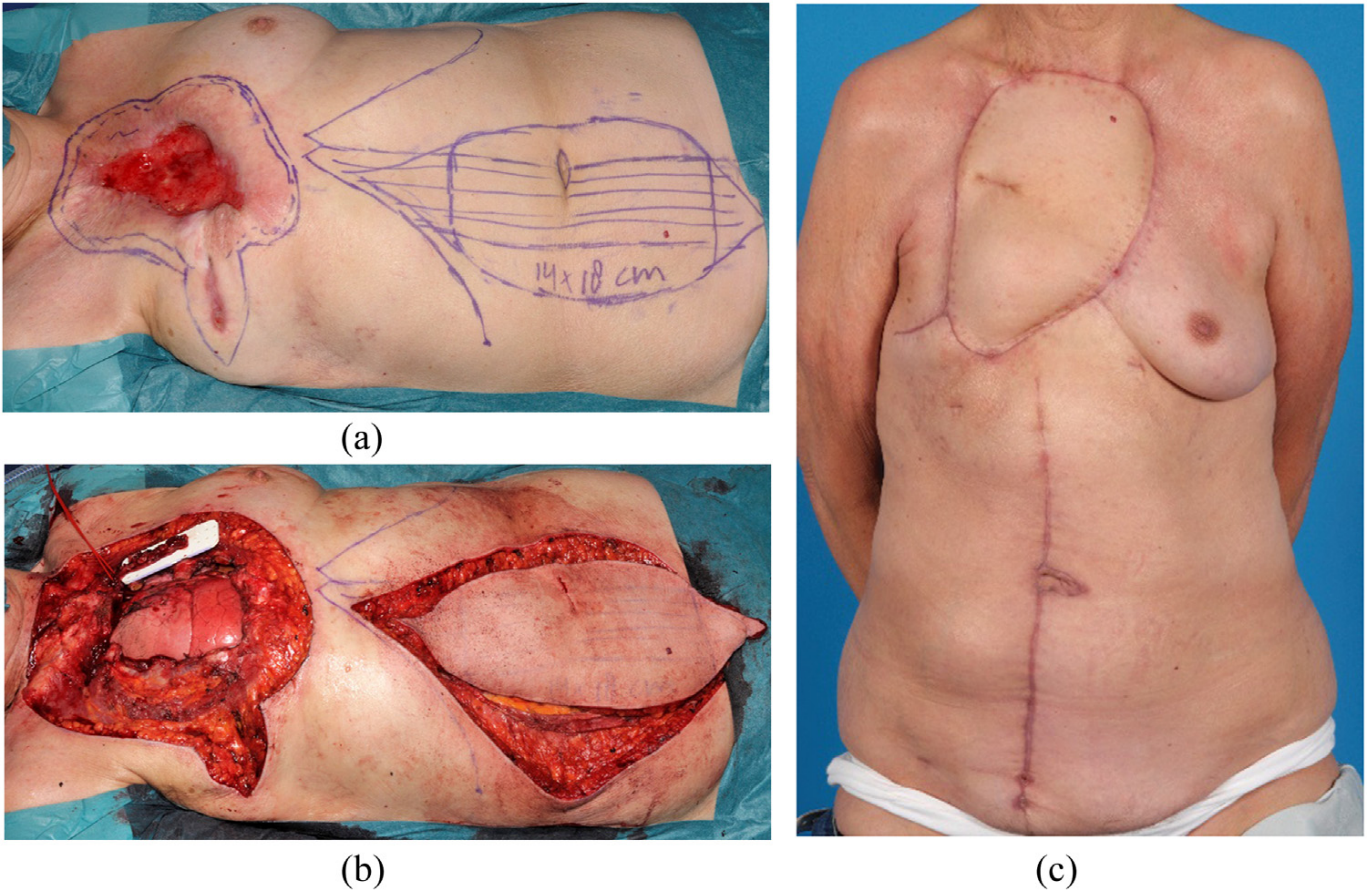
Case with a locally advanced basal cell carcinoma which required subtotal sternectomy and reconstruction using a prolene mesh and free vertical rectus abdominis muscle flap. A. Preoperative planning. B. Intraoperative. C. Postoperative.

Intra- and postoperative photographs are shown in Figure 1, Figure 2 to illustrate two typical cases. In Figure 1 a case is shown with locally recurrent breast cancer, which was resected and reconstructed using a prolene mesh and a pedicled musculocutaneous LD flap. In Figure 2, a case is depicted with a locally advanced basal cell carcinoma which required subtotal sternectomy and reconstruction using a prolene mesh and free vertical rectus abdominis myocutaneous flap.

The median surface area of the defect did not differ significantly between the reconstruction methods with 235 cm^2^ (IQR, 140–290 cm^2^) in free flaps, 186 cm^2^ (IQR, 93–302 cm^2^) in pedicled muscle flaps, 95 cm^2^ (46–118 cm^2^) in muscle advancement flaps and 68 cm^2^ (32–143 cm^2^) in defects without soft tissue reconstruction (*H*=8.949, *p*<0.062). In addition, patients who underwent chemotherapy did not have significantly smaller median defect surface area (110 cm^2^; IQR, 63-201 cm^2^) compared to those who did not receive chemotherapy (180 cm^2^; IQR, 79-273 cm^2^; *U*=155, *p*=0.558).

In total, 22 patients (52.4%) suffered from at least one postoperative complication (Table 4). Two patients had more than one complication. Most of them were wound (21.4%) and pulmonary complications (19.0%). Eight patients (19.0%) had complications with a Clavien–Dindo classification of ≥ 3, which were considered major complications. Two mesh-related complications were encountered in patients who underwent skeletal reconstruction using prosthetic material. One older female patient with osteoporosis showed dehiscence of the implanted prolene mesh resulting in a flail chest after resection of a mesothelioma, including two ribs with a lateral thoracotomy without additional soft tissue reconstruction. This patient required reoperation in which the prolene mesh was replaced by a new one. Another patient developed a fistula to the implanted mesh, which was surgically treated more than 90 days after the reconstruction. One out of eight patients (12.5%) with a free flap reconstruction suffered from total flap failure, which was successfully salvaged with a free anterolateral thigh flap (Table 4).

**Table 4:** Operation characteristics and postoperative complications.

		Total (n = 42)	Missing
Median operative time (IQR), min		229 (147 – 321)	10 (23.8%)
Median defect surface area (IQR), cm^2^		120.0 (72.0 – 255.0)	2 (4.8%)
Complications*		22 (52.4%)	
	Wound complications	9 (21.4%)	
	*Wound infection*	6	
	*Haematoma/Seroma*	2	
	*Dehiscence*	1	
	Pulmonary	8 (19.0%)	
	*Pneumonia*	3	
	*Empyema*	3	
	*Haemothorax*	1	
	*Bronchocutaneous fistula*	1	
	Mesh-related complications	2 (6.1%)	
	*Dehiscence*	1	
	*Fistula*	1	
	Free flap failure	1 (12.5%)	
	Non-surgical complications	4 (9.5%)	
	*New onset atrial* *fibrillation*	1	
	*Acute kidney* *insufficiency*	1	
	*Urinary tract infection*	1	
	*General malaise*	1	
Clavien–Dindo Classification	Grade I	4	
	Grade II	10	
	Grade IIIa	3	
	Grade IIIb	4	
	Grade IVa	1	
	Grade IVb	0	
	Grade V	0	
Median hospital stay (IQR), days		7.0 (5.0 – 10.0)	
Readmission		6 (14.3%)	
Reoperation		5 (11.9%)	
Ninety-day mortality		0 (0.0%)	

⁎ Two patients had >1 complications; 22 patients suffered from 24 complications in total. IQR, interquartile range Clavien–Dindo classification: Grade I; Any deviation from the normal postoperative course without the need for pharmacological treatment or surgical, endoscopic and radiological interventions. Allowed therapeutic regimens are: drugs, such as anti-emetics, antipyretics, analgesics, diuretics and electrolytes, and physiotherapy. This grade also includes wound infections opened at the bedside. Grade II; Requiring pharmacological treatment with drugs other than those allowed for grade I complications. Blood transfusions and total parenteral nutrition are also included. Grade IIIa; Requiring surgical, endoscopic or radiological intervention not under general anesthesia. Grade IIIb; Requiring surgical, endoscopic or radiological intervention under general anesthesia. Grade IVa; Life-threatening requiring IC/ICU-management due to single organ dysfunction. Grade IVb; Life-threatening requiring IC/ICU-management due to multi organ dysfunction. Grade V; Death of a patient.

The median hospital stay was 7 days (Table 4). The overall readmission rate was 14.3%. Reasons for readmission were pneumonia, empyema, bronchocutaneous fistula, wound infection, fistula to implanted mesh and general malaise with vomiting. The overall reoperation rate was 11.9%. Reasons for reoperation were wound infection, mesh dehiscence, empyema, bronchocutaneous fistula and free flap failure. The patient with the bronchocutaneous fistula had a concomitant empyema. Extensive debridement, removal and replacement with a new mesh were necessary in both patients requiring reoperation for empyema and bronchocutaneous fistula. There was no mortality within 90 days after surgery and no hospital mortality.

The only variable significantly associated with the occurrence of any postoperative complication was preoperative chemotherapy (Table 5). Patients who received preoperative chemotherapy showed lower risk for developing postoperative complications (OR=0.182, *p*=0.013). However, the risk of a major complication was comparable between patients with and without preoperative chemotherapy (OR=0.538, *p*=0.689; **Supplementary table 1**). Patients who received chemotherapy did not significantly differ in age, BMI, DM, smoking or ASA classification. There was no association between any of the other variables with major complications.

**Table 5:** Univariable analysis using logistic regression of postoperative complications.

			Postoperative complication N = 22	No postoperative complication N = 20	*p* value	OR 95% CI
Sex	Male		7 (31.8%)	3 (15.0%)	0.284	2.644 0.578–12.095
	Female		15 (68.2%)	17 (85.0%)		
Age, mean (SD), years			63.1 (11.4)	55.7 (13.0)	0.056	NA
BMI, median (IQR), kg/m^2^			25.3 (22.6 – 26.9)	23.5 (22.3 – 26.1)	0.580	NA
ASA score	I–II		16 (72.7%)	17 (89.5%)	0.249	0.314 0.055–1.787
	III–IV		6 (27.3%)	2 (10.5%)		
Diabetes mellitus	Yes		0 (0.0%)	2 (10.0%)	0.221	0.164 0.007–3.643
	No		22 (100.0%)	18 (90%)		
Smoking	Yes		4 (19.0%)	3 (17.6%)	1.000	1.098 0.210–5.750
	No		17 (81.0%)	14 (82.4%)		
Neoadjuvant treatment	Radiotherapy	Yes	5 (22.7%)	2 (10.0%)	0.414	2.655 0.457–15.521
		No	17 (77.3%)	18 (90.0%)		
	Chemotherapy	Yes	4 (18.2%)	11 (55.0%)	**0.013**	0.182 0.045–0.735
		No	18 (81.8%)	9 (45.0%)		
	Chemoradiotherapy	Yes	5 (22.7%)	3 (15.0%)	0.700	1.667 0.343–8.103
		No	17 (77.3%)	17 (85.0%)		
Surface area, median (IQR), cm^2^			140.0 (48.8 – 235.0)	120.0 (103.5 – 255.0)	0.395	NA
Skeletal reconstruction	Mesh		18 (81.8%)	15 (75.0%)	0.714	0.667 0.151–2.936
	No mesh		4 (18.2%)	5 (25.0%)		
Soft tissue reconstruction	Primary closure		4 (18.2%)	3 (15.0%)	1.000	1.259 0.245–6.473
	Soft tissue reconstruction		18 (81.8%)	17 (85.0%)		

SD, standard deviation; IQR, interquartile range; BMI, body mass index; ASA, American Society of Anesthesiologists.

## Discussion

The present study showed that patients undergoing chest wall reconstruction for full thickness defects after oncological resection have high risk of postoperative complications. Wound and pulmonary complications are the most common. However, the reoperation rate of 11.9% was relatively low considering that half of all patients experienced a postoperative complication and that only non-rigid prosthetic materials were used. No postoperative mortality occurred.

Nowadays, due to advancements in anesthesia, imaging studies and reconstruction techniques, chest wall invasion of malignant tumors is rarely a contraindication for surgical treatment. In case of large full thickness chest wall defects, synthetic, biological or composite meshes can be used, with or without titanium plates, to restore thoracic cage rigidity.^20^, ^21^, ^22^ None of these techniques have proven to be superior.^6^^,^^12^ Considering the number of available materials, a wide variety of reconstruction techniques have evolved. The decision on type of reconstruction used depends on size and location of the defect and is usually at the surgeon's discretion. In the present study, only non-rigid prosthetic materials were used for relatively large chest wall defect with median surface area of 120 cm^2^. Among the 33 patients in whom the prosthetic material was used for skeletal reconstruction, only one patient suffered from a flail chest due to mesh dehiscence which was salvaged with replacement of a new mesh. The use of non-rigid prosthetic materials for the reconstruction of full thickness chest wall defects appeared to be adequate in preserving chest wall stability, thereby questioning the need for more rigid fixation techniques.

Evidence-based knowledge on postoperative outcomes after full thickness chest wall reconstruction remains scarce, as chest wall resections are rare. The present study investigated postoperative outcomes after chest wall reconstructions with only non-rigid materials in a homogenous sample of patients who underwent large full thickness chest wall resection. Most previous studies were based on partial and full thickness chest wall reconstructions without reporting the outcomes separately.^1^^,^^6^^,^^9^^,^^10^^,^^13^^,^^14^^,^^16^ In addition, the present study was performed by a multidisciplinary team consisting of a surgical oncologist, cardiothoracic surgeon and plastic surgeon whereas most previous studies were based on a single-surgeon's practice.

The complication rate of 52.4% is comparable to those of previous studies on full thickness chest wall reconstruction, varying from 46.2% to 77.0%.^11^^,^^12^^,^^23^, ^24^, ^25^ The most recent study from 2021 was by Prisciandaro et al. who described a retrospective cohort of chest wall resections for sarcomas followed by reconstruction using rigid and non-rigid materials and reported the highest complication rate of 77%.

The study by Corkum et al. showed a complication rate of 62% in 59 patients after full thickness chest wall reconstruction.^11^ This retrospective analysis further showed that resection of the upper six ribs was associated with general and pulmonary complications. In addition, they reported shorter overall survival after massive chest wall defects in patients who received preoperative chemotherapy, whereas older studies did not find this association.^11^^,^^26^^,^^27^ In our current study, preoperative chemotherapy was paradoxically associated with less postoperative complications. As preoperative chemotherapy aims to decrease the tumor size, it might have been a confounding factor for smaller tumors and resections with less risk of postoperative morbidity. However, the surface area of the defect was not significantly smaller in patients who underwent preoperative chemotherapy. The association found could have been the result of confounding by indication as frail patients with a poor performance status are more likely to be withheld from preoperative chemotherapy, while they are at higher risk for postoperative complications. However, this confounding could not be demonstrated by our data, because patients who received chemotherapy did not differ in age, BMI, DM, smoking or ASA classification from patients who did not receive chemotherapy.

In 1999, Deschamps et al. reported a complication rate of 46.2% in 197 patients who underwent full thickness chest wall resection and reconstruction using only non-rigid materials^12^. Finally, Sabaratnam et al. investigated a cohort of 49 patients who underwent full thickness chest wall reconstruction for primary malignant chest wall tumors using non-rigid materials. They reported a mortality rate of 2% without separately reporting on postoperative complications.

Although half of our patients suffered from at least one postoperative complication, there was no short-term mortality. This is comparable to the results of a previous studies, including partial and full thickness chest wall reconstructions with mortality rates varying from 0.0 to 8.5%.^1^^,^^6^^,^^8^, ^9^, ^10^, ^11^, ^12^, ^13^ Furthermore, in the present study only 11.9% of all patients required reoperation, demonstrating that despite a high total complication rate, full thickness chest wall reconstructions are challenging but safe with a low risk of reoperation and mortality.

The frequent occurrence of wound and pulmonary complications was not an unexpected finding, given the interruption of normal pulmonary physiology following full thickness chest wall reconstruction.^28^ In a study involving 427 patients over a 15-year period, Spacer et al. also found that pulmonary and wound complications were the most frequent after full and partial thickness chest wall reconstructions.^29^

Prehabilitation with exercise programs, smoking cessation programs and nutritional status enhancement may improve postoperative outcomes and reduce complications after chest wall surgery.^30^^,^^31^ Unfortunately, we could not investigate the possible associations between prehabilitation measures and postoperative complications due to a lack of data. Further research into this area is necessary, because prehabilitation may also have the additional benefit of enabling patients deemed unsuitable for surgery to be optimized in such a way that they can undergo extensive chest wall surgery.

The present study has several limitations and the results of our study must be considered in view of these limitations. The accuracy of this retrospective cohort study relies on accurate documentation and retrieval of data on risk factors and outcomes. The limited sample size of our study did not enable multivariable regression analyses. Consequently, it was not possible to determine the independent risk factors for postoperative complications. Therefore, a prospective study is needed to confirm the results of the present study.

## Conclusions

Although more than half of all patients developed postoperative complications after full thickness chest wall resection followed by immediate reconstruction, reconstruction of full thickness chest wall defects using non-rigid materials appears to be safe with an acceptable reoperation rate and low mortality. Therefore, this study showed that the use of non-rigid skeletal reconstruction is adequate in preserving chest wall stability even after extensive full thickness chest wall resections, questioning the need for rigid fixation techniques.

## Appendix Supplementary materials

Image, application 1
