# Supplementary material for: Surgical Outcomes after Full Thickness Chest Wall Resection Followed by Immediate Reconstruction: A 7-Year Observational Study of 42 Cases
Source: JPRAS Open. 2024 Apr 18;41:14–24. doi: 10.1016/j.jpra.2024.04.006 (PMC11153933; doi:10.1016/j.jpra.2024.04.006)
Supplement: Supplementary file 1 [file mmc1.docx]

**Supplementary Table 1.** Univariable analysis using logistic regression of major postoperative complications. Major complication was defined as Clavien–Dindo ≥ 3

|  | |  | Major complication  N = 8 | No major complication  N = 34 | *p* value | OR  95% CI |
| --- | --- | --- | --- | --- | --- | --- |
| Sex | Male |  | 3 (37.5%) | 7 (20.6%) | 0.369 | 2.314  0.442 – 12.114 |
|  | Female |  | 5 (62.5%) | 27 (79.4%) |  |  |
|  |  |  |  |  |  |  |
| Age, mean (SD), years |  |  | 60.6 (19.3) | 63.0 (15.0) | 0.949 | NA |
|  |  |  |  |  |  |  |
| BMI, median (IQR), kg/m^2^ |  |  | 24.4 (20.3 – 27.6) | 25.3 (22.8 – 29.0) | 0.321 | NA |
|  |  |  |  |  |  |  |
| ASA score | I–II |  | 6 (75.0%) | 27 (81.8%) | 0.642 | 0.667  0.107 – 4.150 |
|  | III–IV |  | 2 (25.0%) | 6 (18.2%) |  |  |
|  |  |  |  |  |  |  |
| Diabetes mellitus | Yes |  | 0 (0.0%) | 2 (5.9%) | 1.000 | 0.764  0.033 – 17.473 |
|  | No |  | 8 (100%) | 32 (94.1%) |  |  |
|  |  |  |  |  |  |  |
| Smoking | Yes |  | 2 (25.0%) | 5 (16.7%) | 0.624 | 1.667  0.258 – 10.774 |
|  | No |  | 6 (75.0%) | 25 (83.3%) |  |  |
|  |  |  |  |  |  |  |
| Neoadjuvant treatment | Radiotherapy | Yes | 3 (37.5%) | 4 (11.8%) | 0.113 | 4.500  0.766 – 26.451 |
|  |  | No | 5 (62.5%) | 30 (88.2%) |  |  |
|  |  |  |  |  |  |  |
|  | Chemotherapy | Yes | 2 (25.0%) | 13 (38.2%) | 0.689 | 0.538  0.094 – 3.078 |
|  |  | No | 6 (75.0%) | 21 (61.8%) |  |  |
|  |  |  |  |  |  |  |
|  | Chemoradiotherapy | Yes | 0 (0.0%) | 8 (23.5%) | 0.316 | 0.183  0.010 – 3.522 |
|  |  | No | 8 (100%) | 26 (76.5%) |  |  |
|  |  |  |  |  |  |  |
| Surface area, median (IQR), cm^2^ |  |  | 185.0 (80.9 – 251.1) | 115.5 (72.0 – 255.0) | 0.578 | NA |
|  |  |  |  |  |  |  |
| Skeletal reconstruction | Mesh |  | 7 (87.5%) | 26 (76.5%) | 0.662 | 2.154  0.229 – 20.235 |
|  | No mesh |  | 1 (12.5%) | 8 (23.5%) |  |  |
|  |  |  |  |  |  |  |
| Soft tissue reconstruction | Primary closure |  | 1 (12.5%) | 6 (17.6%) | 1.000 | 0.667  0.069 – 6.474 |
|  | Soft tissue reconstruction |  | 7 (87.5%) | 28 (82.4%) |  |  |

SD, standard deviation; IQR, interquartile range; BMI, body mass index; ASA, American Society of Anesthesiologists.
